# Crude Ethanol Extract of *Pithecellobium ellipticum* as a Potential Lipid-Lowering Treatment for Hypercholesterolaemia

**DOI:** 10.1155/2014/492703

**Published:** 2014-04-15

**Authors:** Janet P.-C. Wong, Sumi Wijaya, Kang-Nee Ting, Christophe Wiart, Kamarul'Ain Mustafa, Fiona Shipton, Teng-Jin Khoo

**Affiliations:** ^1^Center for Natural and Medicinal Products Research, School of Pharmacy, Faculty of Science, University of Nottingham, Malaysia Campus, Jalan Broga, 43500 Semenyih, Selangor, Malaysia; ^2^School of Biomedical, Faculty of Science, University of Nottingham, Malaysia Campus, Jalan Broga, 43500 Semenyih, Selangor, Malaysia; ^3^Faculty of Chemistry, Universiti Sultan Zainal Abidin, Jalan Sultan Mahmud, 20400 Kuala Terengganu, Malaysia

## Abstract

If left untreated, hypercholesterolaemia can lead to atherosclerosis, given time. Plants from the Fabaceae family have shown the ability to significantly suppress atherosclerosis progression. We selected four extracts from *Pithecellobium ellipticum*, from the Fabaceae family, to be screened in a 3-hydroxy-3-methylglutaryl coenzyme A reductase (HMG-CoA reductase) assay. The ethanol extract, at a concentration of 500 **μ**g/mL, exhibited superior inhibition properties over the other extracts by demonstrating 80.9% inhibition, while 0.223 **μ**g/mL of pravastatin (control) showed 78.1% inhibition towards enzymatic activity. These findings led to the fractionation of the ethanol extract using ethyl acetate : methanol (95 : 5), gradually increasing polarity and produced seven fractions (1A to 7A). Fraction 7A at 150 **μ**g/mL emerged as being the most promising bioactive fraction with 78.7% inhibition. FRAP, beta carotene, and DPPH assays supported the findings from the ethanol extract as it exhibited good overall antioxidant activity. The antioxidant properties have been said to reduce free radicals that are able to oxidize lipoproteins which are the cause of atherosclerosis. Phytochemical screenings revealed the presence of terpenoid, steroid, flavonoid, and phenolic compounds as the responsible group of compound(s), working individually or synergistically, within the extract to prevent binding of HMG-CoA to HMG-CoA reductase.

## 1. Introduction

Hypercholesterolaemia is the main contributor to numerous cardiovascular diseases (CVD) with substantial data from studies to support this claim [1, 2]. It is predicted that over a billion deaths will be due to CVD by the earlier half of the 21st century [3]. Mackey and Co. (2007) estimated that approximately 18.1 million deaths were due to cardiovascular disease in 2010 and that we will see an increase of 33.7% by 2030.

Hypercholesterolaemia can lead to atherogenesis. Atherosclerosis is the primary risk factor for coronary heart disease and affects the peripheral arteries and cerebral circulation. Studies relating to cancer and total cholesterol levels have shown that the total cholesterol level is the main risk factor for disease. However, most of the cholesterol is carried in the plasma (60–70%), in the form of low density lipoproteins (LDLs) and it is this which is the main risk factor for disease [4, 5].

Overproduction of reactive oxygen species has been strongly associated to the development of oxidation related conditions like atherosclerosis and cardiovascular diseases [5, 6]. Atherosclerosis begins with the transmigration of oxidized LDLs to the intima (the subendothelial space) which cause injuries to endothelial cells. The injury is described as cellular damage and one result of this type of damage is the loss of function of the cell [4, 7, 8]. Endothelial cells respond to the injury by becoming sticky which alerts the macrophages to ingest the oxidized LDLs. These macrophages are then overloaded with oxidized LDLs; the appearance of the lipids in the macrophages gives them a foamy appearance; hence they are given the name “foam cells.” The accumulation of foam cells leads to the formation of fatty streaks [7]. After some time atheroma takes place, the smooth muscle cells migrate and further restrict the blood flow leading to atherosclerosis. Complex lesions occur with the advancement of inflammatory responses, necrotic core formation, and death of foam cells. Finally, thrombosis occurs when fibrous caps rupture [4, 7–9]. 3-Hydroxy-3-methylglutaryl coenzyme A reductase (HMG-CoA reductase) is the rate limiting factor for the formation of mevalonate in the cholesterol biosynthesis pathway. This enzyme has been chosen as one of the pharmacological targets to control the production of cholesterol* de novo*. Statins are widely used to treat hypercholesterolaemia because of their ability to exhibit superior suppression of lipid synthesis at a nanomolar scale [10]. The hydrophobic group of a statin is suggested to be the core reason for this nanomolar inhibition constant value, *K*_*i*_ ~ 1 × 10^−9^ M, while the substrate's *K*_*m*_ value is ~10^−5^ M [10, 11]. This gives the enzyme a 10,000-fold higher binding affinity towards statins compared to the substrate [10].

Generally, a statin's heptatonic acid functional group resembles the HMG moiety of the substrate at the molecular level. Istvan and Deisenhofer (2001) proved that the heptatonic acid can be easily competed with and bound at the O5-hydroxyl group of the HMG-like moiety and that it replaced the thioester oxygen atom of the substrate. The conformational flexibility has sterically hindered the substrate from binding with the enzyme. Concurrently, the statin structure extends into the narrow pantothenic acid-binding pocket moiety of coenzyme A which eventually blocks the coenzyme A of the substrate [11, 12]. This is why statins are capable of competing with the substrate.

Besides effectively interfering with cholesterol biosynthesis via inhibiting the reductase enzyme, statins are gaining attention for their antiatherosclerosis effect due to their antioxidant ability. A few of the statins' antioxidant defense mechanisms include reducing lipoprotein cholesterol which reduces substrate oxidation, attenuating development of superoxide anions in endothelial cells to prevent oxidation of LDLs and preventing diffusion of free radicals produced from oxidative stress towards the lipoprotein core [13]. Fluvastatin sodium has shown such ability [14].

Currently available cholesterol lowering drugs such as statins do pose a risk of myopathy by depressing ubiquinone Q_10_ biosynthesis, rhabdomyolysis, and other considerable side effects like comorbidities and several noncardiac effects (gratuitous thoughts, short temper), psychiatric events, and even impairment of the central nervous system [15, 16]. This opens a need for more research into natural product such as plants from the Fabaceae family [2].* Pithecellobium* sp. shrubs and trees are distributed in the secondary forests and riverbanks of Tropical Asia and Subtropics of America [17–22]. Approximately 200 species have been identified from this genus [23]. This genus exhibited antifungal [24–27], antibacterial [25, 27], anti-inflammatory [28], antiallergic effects [28], insect antifeedant [29], piscicide [30], and antioxidant activity [31–34].

*Pithecellobium ellipticum* is also known as* Archidendron ellipticum* (Blume) I. C. Nielsen and commonly called kabau (*Jering tupai*) in Malaysia [22]. Wild fruits of* P. ellipticum* are enjoyed by Malaysians in salad and cooking due to their delicious flavor [10]; they are used as an astringent in Indonesia [21], as a remedial shampoo in Java, and as fish poison [35]. This particular plant has not been thoroughly studied for its lipid-lowering properties. A* T*.* indica* ethanol extract from the same family has been reported to decrease non-HDL cholesterol by 73%, triglycerides by 60%, and even increase HDL cholesterol up to 61% [2, 9]. Besides this, radical scavenging tests disclosed that in some* in vivo* studies the extract improved antioxidant abilities in an animal model [9]. These findings indicate that the properties within the extract are capable of reducing atherosclerosis. The aim of this study is to assess the HMG-CoA reductase inhibition ability, antioxidant properties, and screening for the presence of secondary metabolites from the crude extract of the leaves of* P. ellipticum* (code: UNMC 35L).

## 2. Results and Discussion

All of the extracts prepared only with the extract and buffer showed absorption at 340 nm in the spectrophotometric analysis (results not shown). This is not a surprise as crude extracts are expected to contain numerous compounds. Therefore, all extracts require colour correction to ensure all possible interfering absorbances contributed by the extracts in the HMG-CoA reductase assay are minimized. This is also important as NADPH molecules absorb light at the same wavelength and their oxidized form NADP^+^ does not [36]. This is the reason why this enzymatic assay was analyzed at 340 nm and requires minimal interference at 340 nm from the samples analyzed.

Table 1 shows the specific activity and percentage of inhibition of all the crude extracts. Any specific activity obtained lower or near the pravastatin range is considered to have the ability to inhibit HMG-CoA reductase activity. Values near or above the negative control's specific activity implies that the particular sample either is a very weak inhibitor or may elevate the enzymatic activity. The ethanol (E) extract stood out as the extract with the most potential followed by the water (W), ethyl acetate (EA), and finally hexane (H) extract with the least inhibitory capability. The specific activity of the E extract was calculated as being 21.8 times lower than that of the H extract, making it a superior inhibitor to the H extract.

**Figure 1:**
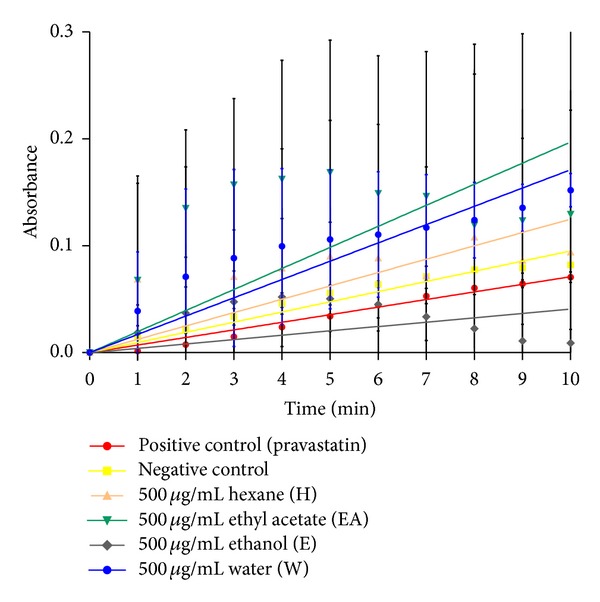
HMG-CoA reductase specific activity of crude extracts.

The difference in inhibition competency between E and W extracts was 17.8%. This might be due to the concentration of active compounds present within the E extract being greater or that the competition for the active moiety favors the E extract over the W extract. Another probability would be the binding locations; bonds and presence of inhibitor active moiety involved in the inhibitor-enzyme complex are sturdier and not easily broken. For example, the constituents in the E extract might have attached firmly at the active pocket, while constituents within the W extract bind weakly instead. Additionally, a different pharmacological activity is contributed by an inhibitor's active moiety, such as that which is present in Pitavastatin [37]. The inhibition value of the E extract at 80.9% was comparable to pravastatin (78.1%) even though the concentration used was distinctively huge. It is essential to realise that even though 500 *μ*g/mL is considered a high concentration, this is the concentration of the crude extract and the active compound within the extract is hypothesized to express this suppression ability at a much lower concentration. Another key finding from Figure 1 is that the W extract which appeared to have some suppressing activity (0.503 units/mg protein) became noticeably weaker over the duration of 10 minutes. The line graph of the W extract is above the negative control line on the graph. This means that the concentration of the active compound within the W extract is exhausted and the activity is reversible, allowing the natural substrate to take over once again and bind with the HMG-CoA reductase.

**Figure 2:**
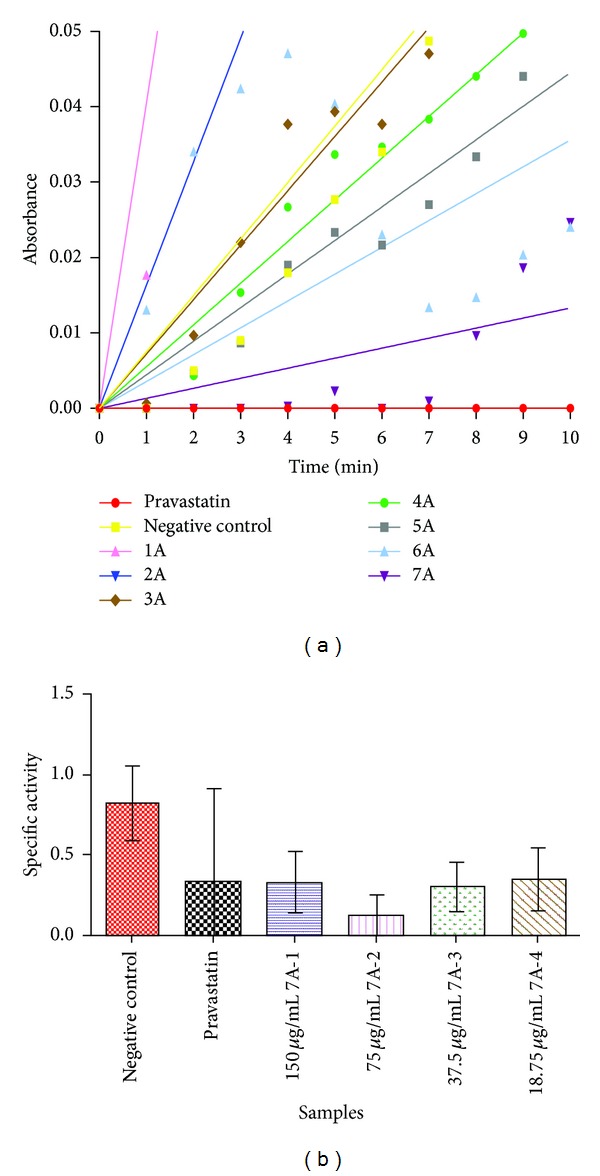
(a) HMG-CoA reductase specific activity on 35L E 1A–7A fractions; (b) HMG-CoA reductase specific activity on UNMC 35L 7A twofold dilution. There is no significant difference with *P* < 0.05 in Tukey's multiple comparison tests. Results are demonstrated as average ± standard deviation (*n* = 2 : 3 different experiments).

The finding attained from the extract inhibitory screening has led to the fractionation of the ethanol extract as it had the highest suppressing strength. The resulting fractionation produced 7 fractions labeled 1A–7A. Fractionation was carried out with increasing solvent polarity. Fraction 7A (consisting of the last thirteen vials from fractionation) was found to be the most promising fraction in influencing the HMG-CoA reductase activity (Table 2 and Figure 2).

Fraction 7A had the highest inhibition activity (78.7%) with a specific activity of 0.211 ± 0.2 units/mg protein (Figure 2(a) is expended for viewing purpose). Twofold dilutions of 7A were prepared to reveal the effectiveness of this fraction. It showed an apparent trend with increasing solvent polarity used for fractionation; the fractions achieved higher inhibitory properties as seen from fractions 3A to 7A. In contrast, 5A did not comply with the trend by disclosing only 16.3% inhibition.

Statistical analysis indicated that twofold dilution samples of 7A labeled 7A-1 to 7A-4 actually have no significant difference between them or pravastatin (Figure 2(b)), but pravastatin concentration was kept at 0.223 *μ*g/mL. Despite this, mL7A-2 exhibited higher inhibition activity compared to 7A-1 which was not expected. 7A-3 and 7A-4 fractions did not express any distinct difference to 7A-1. Six different phytochemicals, including saponins, flavanoids, tannins, phenols, terpenoids, and steroids, were screened for, to find out which secondary metabolites were present. Phytochemical screening results for* P. ellipticum* were in agreement with the isolation of saponins from leaves that was reported earlier by Beutler et al. [18]. The hexane extract consisted of flavanoids and terpenoids, while the EA extract had tannins, terpenoids, and steroids. The E extract had positive reactions in all tests except for tannins. Meanwhile, flavanoids and steroids were absented in the W extract. There is a possibility that the flavonoid, phenolic content, terpenoid, and steroid compounds are responsible (Table 3) for effective competition with the substrate, for binding at or blocking the substrate from binding at the enzyme's narrow pocket moiety. Saponins are an exception in this enzymatic discussion because saponins are categorized as a bile acid sequestrant that works within the intestines [38]. Thus, this eliminates the possibility of saponins suppressing the reductase activity in the inhibitory assay that was conducted. Saponins have been reported to elevate the activity and expression of hepatic lipase located at the liver and inhibit the mRNA and expression of fatty acid synthase to reduce lipids in a hyperlipidaemic animal model [38].

Lipid peroxidation involves the forming and spreading of reactive oxygen species (ROS). Lipid hydroperoxides are produced in the presence of oxygen in lipid oxidation. ROS lead to oxidative stress that also induces atherosclerosis and which makes the antioxidant analyses a very important part of our investigation, especially in the case of the E extract in determining whether it truly possesses antioxidant properties. Three antioxidant assays, FRAP, *β*-carotene bleaching assay, and DPPH, were conducted on the crude extracts and recorded in Table 4. Lipid peroxidation began with Fe^3+^ and tripyridyl triazine (TPTZ). Extracts with antioxidant properties successfully reduce Fe^3+^ to Fe^2+^. With regards to the FRAP assay values, the E extract had the highest antioxidant activity with 0.843 mmol FeSO_4_ equivalent/L and followed by the EA, W, and lastly H extract.

Inhibition of oxidation in the *β*-carotene bleaching assay samples are arranged in decreasing order: W > H > E > EA. EC_50_ of the W extract was 4.631 × 10^−5^ *μ*g/mL which appeared to be more effective than quercetin and only two times weaker than Trolox. This reveals that the potential antioxidant compound isolated from the W extract might be even more potent than Trolox. Both H and extracts however demonstrated roughly similar abilities in protecting the *β*-carotene compound; meanwhile the H extract proved to be a poorer antioxidant extract in the FRAP assay with 16.96 times lower antioxidant activity than the E extract.

DPPH results have wider IC_50_ values ranging between 460.930 and 12.738 *μ*g/mL. The EA extract showed the greatest free radical scavenging activity with an IC_50_ of 12.738 *μ*g/mL and this value does not differ much from that of the E extract (approximately 1.09 times more powerful). Trolox (1.09 *μ*g/mL) was 12.7 times stronger than the E extract. The H extract was the least potent of all (IC_50_ = 460.930 *μ*g/mL). The ethyl acetate and ethanol extracts were ranked as the most and second potent-free radical scavengers, respectively, while the W extract belongs to the intermediate antioxidant group obtaining an IC_50_ of 145.403 *μ*g/mL. The H extract had the lowest free radical quenching capacity. An interesting note is that the E extract was the most potent inhibitor in the HMG-CoA reductase assay and has consistently exhibited strong antioxidant activity in the three assays conducted. However, other extracts demonstrated potent antioxidant activity in certain assays but not in others. No consistently low values were found in the assays for the poorly active extracts except for the H extract which was the weakest in both FRAP and DPPH assays. The ethyl acetate extract had the greatest activity in both FRAP and DPPH assays meanwhile the W extract was, strongest in the *β*-carotene bleaching assay.

Their antioxidant properties are expected to correlate with the results of the phytochemical screenings. This would be due to phenolic compounds and their derivatives that are capable of preventing auto-oxidation. Phenolic compounds are bioactive substances that are recognized for their antioxidant ability. From the correlation of results, components from 7A fraction might contain antioxidant properties in addition to its enzyme inhibition ability.

This antioxidant compound from the active extracts may potentially be capable of affecting both free radicals from causing oxidative stress and the enzyme activity, reducing atherosclerosis progression in hypercholesterolaemic patients as statins do. Statins have been tested in various antioxidant assays to demonstrate their ability in reducing atherosclerosis in ways other than directly inhibiting HMG-CoA reductase [39, 40]. The antioxidant compound(s) from this plant that is responsible for interrupting HMG-CoA reductase in the assay might behave similar to statins. Reduction of Fe^3+^-TPTZ complex to Fe^2+^-TPTZ complex could be caused by chain termination of peroxyl and hydroxyl radical formation caused by the antioxidant compounds, especially in the active extract. Lovastatin and simvastatin have been shown to stimulate formation of Fe^2+^ [39].

Statins which are synthesized or naturally isolated have demonstrated different lipid peroxidation and scavenging activity. Another possibility would be that the functional group present within the compound itself from* P. ellipticum* exerts an antioxidant ability like in the case of the enol moiety conjugated to the indole ring of fluvastatin sodium [14]. The hydrophobic side chain of the potential active compound is a contributing factor to the potency of the binding affinity towards HMG-CoA reductase. Furthermore, the same antioxidant compound could also donate an electron to the free radicals through scavenging activity as reported in the cases of simvastatin and rosuvastatin [39].

## 3. Materials and Methods

### 3.1. Plant Material and Extraction Procedure

Leaves of* P. ellipticum* were collected from Sungai Congkak Forest, Malaysia. The plant authenticity was verified and herbarium specimen deposited at Forest Research Institute Malaysia (FRIM) with voucher number of PID160610-06. A portion of plant material (232 g) was macerated with hexane overnight in a ratio of 1 : 8 (plant material : solvent) and extracted three times. This extraction process was subsequently repeated using ethyl acetate, ethanol, and finally water. Combined extracts yielded H (0.46%), EA (1.48%), E (1.72%), and W (4.83%).

### 3.2. Qualitative Analysis by UV-Vis Spectrometer

All the crude extracts were prepared at 500 *μ*g/mL and scanned using PerkinElmer, Lambda 25 model to monitor colour correction at 340 nm. Extracts showing chromophore and auxochrome at 340 nm region were prepared for colour correction.

### 3.3. HMG-CoA Reductase Assay

HMG-CoA reductase assay kit was purchased from Sigma Malaysia. The kinetic activity measurements were carried out in triplicate and determined by employing the spectrophotometric method from Sigma CS 1090 with minor modifications. 500 *μ*g/mL (crude extracts) and 150 *μ*g/mL and its twofold dilutions (fractions) were dissolved in dimethyl sulphoxide, DMSO (Labscan). Assay kit components were incubated for 15 minutes at 37°C prior to analysis. A different amount of 1 X assay buffer was added (blank: 183 *μ*L, inhibitor: 181 *μ*L, activity: 182 *μ*L, samples: 182 *μ*L, and sample correction: 199 *μ*L), 1 *μ*L DMSO was added to the blank, inhibitor, and activity wells, 1 *μ*L of pravastatin into inhibitor wells, 4 *μ*L of NADPH, and 12 *μ*L substrate were added into all wells and incubated for another 5 minutes. Finally, 2 *μ*L of reductase enzyme was added. Samples were prepared in duplicate. The kinetic study was analyzed at 340 nm and at 37°C with the shake mode on for 10 minutes using Varioskan Flash (Thermofisher). Specific activity was expressed in unit/mg protein:

(1) unitmg  protein =(ΔA340/min⁡sample⁡−ΔA340/min⁡control⁡)×TV12.44×V×0.6×LP

12.44 = *ε* ^*nM*^—the extinction coefficient for NADPH at 340 nm is 6.22 m/Mcm. 12.44 represents two NADPH consumed in the reaction. TV: total volume of the reaction in mL (0.2 mL for plates), *V*: volume of enzyme used in the assay (mL), 0.6: Enzyme concentration in mg-protein (mg protein)/mL that is 0.55–0.65 mg protein/mL, and LP: light path in cm (0.55 for plates).

### 3.4. Fractionation of the Ethanol Extract

The ethanol extract (1.4 g) was chosen for further fraction. Silica 60 (Merck, 0.063–0.200 mm) was used as the packing material with the ratio of 1 g sample : 50 g silica. Starting solvent system was ethyl acetate : methanol (95 : 5) increasing the methanol gradually, followed by ethanol, 1% of acetic acid, and finally 2% of acetic acid in ethanol. A total of 113 fractions were collected and combined into seven major fractions (35L E 1A to 7A) after thin layer chromatography profiles were determined with different ratios of chloroform-methanol, ethyl acetate-methanol, and butanol-acetic acid-water solvent systems using detection under UV light, iodine vapor, vanillin, ferric chloride, and antimony (III) chloride spray reagents. All fractions (150 *μ*g/mL and its two-fold dilutions) were later subjected to the HMG-CoA reductase assay to determine the active fractions.

### 3.5. Phytochemical Screenings

Several tests were performed to confirm the presence and identity of secondary metabolites within the crude extracts: saponins, flavonoids, tannins, phenolics, steroids, and terpenoids. Approximately 12.5 mg of each extract was prepared.

#### 3.5.1. Test for Saponins (Frothing Method)

Each extract was dissolved in 5 mL of purified water in the test tube and covered with cork according to method by H. A. Ibrahim and H. Ibrahim. The samples were sonicated for 15 minutes at 40°C. Samples that had particles were filtered before being shaken vigorously for thirty seconds and then left for another 45 minutes. Persistent froth revealed the presence of saponins [41].

#### 3.5.2. Test for Flavonoids (Modified Shinoda Test)

Flavonoid analysis was prepared following the Modified Shinoda Test [42]. All extracts were dissolved in 5 mL of DMSO. 3-4 cm of magnesium turnings and 6 drops of 36% concentrated HCl were added. Various colours such as orange, pink, and red to purple represent flavones, flavonols, 2,3-dihydro derivate, and xanthone, respectively.

#### 3.5.3. Test for Tannins and Phenolic Content (Gelatin Precipitation and Ferric Chloride)

This test by Mojab and colleagues used two reagents: gelatin solution for protein precipitation and ferric chloride to confirm the presence of phenolics. Extracts were dissolved in 5 mL purified water and sonicated for 15 minutes at 40°C. The volume was divided into three portions—one for control, gelatin precipitation, and phenolics. White fog or precipitation with 5 mL of 1% gelatin indicates the presence of tannins, while a change from colourless to brownish-green or a blackish-blue colour after 6 drops of 1% ferric chloride indicates the presence of phenolics [43].

#### 3.5.4. Test for Steroids/Terpenoids (Salkowski Test)

Both steroid and terpenoid tests were accomplished using the Salkowski test [44, 45]. Every extract was dissolved in 5 mL of DMSO and sonicated for 15 minutes at 40°C. One mL of solution was added to 1 mL chloroform and an equal volume of concentrated sulphuric acid was slowly added down the side of the test tube. The upper layer changed to a red colour and the sulphuric acid layer displayed a yellow colour with green fluorescence. A reddish-brown colour observed at the interface corresponded to the presence of terpenoids, while a blue or green interface indicated the presence of steroid compounds.

### 3.6. Antioxidant Assays Evaluation

All crude extracts were dissolved in DMSO. Quercetin and trolox were used as standards.

#### 3.6.1. Ferric Reducing Ability Power (FRAP) Assay

The reagents were prepared according to the reports of Benzie and Strain. 180 *μ*g/mL fresh FRAP was pipetted and followed by 20 *μ*L of the sample (in triplicate). The microplate was incubated for 90 minutes prior to being monitored spectrophotometrically at 593 nm through a Dynex Microplate Reader. Concentrations ranging from 1 to 125 mM of ferric sulphate concentrations were prepared for calibration. FRAP values were expressed as the concentration of the extracts that showed an absorbance value equal to 1 mM of FeSO_4_ [46].

#### 3.6.2. *β*-Carotene Bleaching Method

This experimental procedure was slightly modified from Miller [47]. Two mg of *β*-carotene was dissolved in 10 mL CHCl_3_. Then 2 mL was pipetted into a 100 mL round bottom flask and CHCl_3_ was later removed. Subsequently, 40 mg purified linoleic acid; 400 mg Tween 80 emulsifier; and 100 mL distilled water were added and shaken. Readings were measured at 490 nm with a Dynex Microplate Reader at 20 minute intervals and incubated at 50°C for four hours. Both the blank and wells without *β*-carotene solution were used for background subtraction. The following equations were used to calculate the rate of degradation and antioxidant activity. EC_50_ value calculated represents 50% antioxidant ability:

(2) Sample  degradation  rate=ln⁡⁡(Abst=0Abst=240)×1240,Antioxidant  activity,% =100%×(degradationcontrol−degradationsampledegradationcontrol).

#### 3.6.3. Free Radical Scavenging Activity

Twenty microliters of (0.003 up to 1 mg/mL) samples were added with 180 *μ*L (0.1 mM) DPPH (2,2-diphenyl-1-picrylhydrazyl). Scavenging activity absorbance was performed at 550 nm via Varioskan after incubating for 30 minutes in the dark, at room temperature [48]. The antioxidant activity was presented as the IC_50_ which is 50% of the crude extract concentration required for quenching free radicals. Initially the samples were purple and decoloration to yellow (reduced form of DPPH) disclosed the presence of the scavenging potential of the antioxidant extract [49]:

(3) $$\begin{array}{c} \text{DPPH}\;\text{inhibition},\%=100\times \left(\frac{{\text{Abs}}_{\text{control}}/{\text{Abs}}_{\text{sample}}}{{\text{Abs}}_{\text{control}}}\right). \end{array}$$

### 3.7. Statistical Analysis

The statistical analysis was performed using GraphPad Prism 5.02 Software. The data was compared with analysis of variance, ANOVA, and Tukey Multiple Comparison Test as the posttest. Probability value of *P* < 0.005 was selected for significant difference.

## 4. Conclusions

Through the HMG-CoA reductase assay, the ethanol extract of* Pithecellobium ellipticum* revealed itself to be a promising hypercholesterolaemic lowering agent. The responsible compounds might be flavonoids, phenolics, terpenoids, or even steroids. Negative results in the phytochemical screening might have been due to either the secondary metabolite not being present or being present in a minute amount which could not be detected. Overall, the ethanol extract exhibited good antioxidant ability in the three antioxidant assays. Of course, further tests will be required to confirm whether the active component is also an antioxidant substance. Additional isolations and identifications are being carried out for fraction 7A to find the constituents that are able to influence HMG-CoA reductase activity.

## Figures and Tables

**Table 1:** Percentage of inhibition of UNMC 35L crude extract.

Sample	Specific activity (units/mg protein)	% inhibition
H crude extract^A^	5.668 ± 2.9^a^	0
EA crude extract^A^	3.638 ± 3.0^b^	0
E crude extract^A^	0.260 ± 0.6^ac^	80.9
W crude extract^A^	0.503 ± 0.5^ad^	63.1
Positive control (pravastatin)^B^	0.298 ± 0.3^abe^	78.1
Negative control	1.363 ± 0.5^af^	0

Values with the same letter are significantly different *P* < 0.05 by Tukey's multiple comparison tests.

Results are demonstrated as average ± standard deviation (*n* = 2 : 3 different experiments).

^A^Crude extracts are prepared at a concentration of 500 *μ*g/mL.

^B^Pravastatin is prepared at a concentration of 0.223 *μ*g/mL.

**Table 2:** Percentage of inhibition of UNMC 35L ethanol fractions.

Fractions	Specific activity (units/mg protein)	% inhibition
1A^A^	2.744 ± 0.9	0
2A^A^	0.812 ± 0.6	18.0
3A^A^	0.974 ± 0.6	1.6
4A^A^	0.552 ± 1.0	44.2
5A^A^	0.829 ± 0.6	16.3
6A^A^	0.520 ± 0.4	47.5
7A^A^	0.211 ± 0.2	78.7
Positive control (Pravastatin)^B^	0.195 ± 0.3	100.0
Negative control	1.234 ± 0.8	0

All values are not significantly different *P* < 0.05 by Tukey's multiple comparison tests.

Results are demonstrated as average ± standard deviation (*n* = 2 : 3 different experiments).

^A^1A to 7A fractions are prepared at a concentration of 150 *μ*g/mL.

^B^Pravastatin is prepared at a concentration of 0.223 *μ*g/mL.

**Table 3:** Phytochemical screenings of UNMC 35L crude extracts.

Types of screenings	Leaves extracts			
	H	EA	E	W
Saponins	−	−	+	+
Flavonoids	+	−	+	−
Tannins	−	+	−	+
Phenolic contents	−	−	+	+
Terpenoids	+	+	+	+
Steroids	−	+	+	−

Results expressed as + and – indicate, respectively, the presence and absence of secondary metabolites within the crude extracts.

**Table 4:** Different types of antioxidant analyses.

Crude samples	Type of analysis			
	FRAP		*β*-Carotene	DPPH
	mmol FeSO_4_ equivalent/L	*R* ^ 2^	EC_50_ (*μ*g/mL)	IC_50_ (*μ*g/mL)
Hexane	14.301 ± 0.0^ad^	1.0000	7.188 × 10^−3^ ^a^	460.930 ± 0.2^a^
Ethyl acetate	1.093 ± 0.7^ab^	0.9812	1.775 × 10^−2^ ^a^	12.738 ± 0.4^ab^
Ethanol	0.843 ± 0.1^ac^	0.9453	1.059 × 10^−3^ ^a^	13.830 ± 0.0^ac^
Water	1.240 + 0.3^ad^	0.9964	4.631 × 10^−5^ ^ab^	145.403 ± 0.1^ad^
Quercetin	0.003 ± 0.1^ade^	0.9992	1.132 × 10^−4^ ^ac^	0.110 ± 0.0^ae^
Trolox	0.010 ± 0.0^adf^	0.9906	9.730 × 10^−5^ ^ad^	1.090 ± 0.0^af^

Values in the same column that are followed by the same letter are significantly different *P* < 0.05 by Tukey's multiple comparison tests.

Results are expressed as means ± S.D (*n* = 2 : 3 different experiments).
